# Exclusive Effects of Moxibustion on Gut Microbiota: Protocol for a Focused Systematic Review and Meta-Analysis

**DOI:** 10.2196/73317

**Published:** 2025-10-24

**Authors:** Xiaotao Zhang, Yueyue Guo, Jia Shi, Qianli Zang, Ying Li

**Affiliations:** 1 Jiangsu Province Hospital of Chinese Medicine, Affiliated Hospital of Nanjing University of Chinese Medicine, 200 Xianlin Avenue, Nanjing, 210023, China, 86 18205168666

**Keywords:** moxibustion, gut microbiota, systematic review, meta-analysis, traditional medicine, microbiome

## Abstract

**Background:**

The gut microbiota (GM) plays a critical role in systemic health, influencing immune, metabolic, and neurological functions. There is emerging evidence suggesting that moxibustion, a traditional thermal therapy, may modulate the GM to restore microbial homeostasis, yet its exclusive effects remain undifferentiated from those of combined therapies such as acupuncture. Previous meta-analyses lack mechanistic specificity, necessitating a focused evaluation of moxibustion’s impact on microbial ecology.

**Objective:**

This systematic review and meta-analysis aims to quantify moxibustion-induced changes in GM diversity, taxonomic composition, and functional metabolites (eg, short-chain fatty acids).

**Methods:**

We will systematically search the PubMed, Web of Science, Cochrane Library, China National Knowledge Infrastructure, Wanfang, and VIP databases from inception to December 31, 2024, using keywords such as “moxibustion,” “gut microbiota,” and “intestinal flora.” Eligible preclinical (animal) and clinical (human) studies evaluating stand-alone moxibustion interventions on the GM will be included. Primary outcomes include microbial α diversity indexes (Shannon and Simpson) and relative abundance of key taxa (eg, *Firmicutes* and *Bacteroidetes*). Risk of bias will be assessed using the Systematic Review Center for Laboratory Animal Experimentation risk-of-bias tool for animal studies and the modified Collaborative Approach to Meta-Analysis and Review of Animal Data from Experimental Studies criteria for human trials. Pooled effect estimates for continuous outcomes (eg, diversity indexes and taxa ratios) will be calculated using the ratio of means with 95% CIs. Statistical analyses will be conducted in RevMan (version 5.4) and R (*metafor* package), with data archived on Figshare for reproducibility.

**Results:**

As of March 2025, the literature search and screening have been completed, and 31 studies meeting the inclusion criteria have been identified. The comprehensive analysis is scheduled to be completed by October 2025, with results anticipated to be published in late 2025. On the basis of previous work, an anticipated result is that moxibustion may reduce pathogenic genera such as *Ruminococcus* while enhancing beneficial genera, effects that are expected to be associated with improved intestinal barrier integrity and anti-inflammatory responses.

**Conclusions:**

This protocol provides a rigorous framework to evaluate moxibustion’s unique role in GM modulation, bridging traditional medicine with microbiome science. The results will inform optimized, nonpharmacological strategies for managing microbiome-associated chronic diseases and guide future research priorities.

## Introduction

The gut microbiota (GM) constitutes a diverse and dynamic microbial ecosystem in the gastrointestinal tract, comprising bacteria, archaea, fungi, viruses, and protozoa [1,2]. It is closely linked to host physiology, influencing immune modulation, metabolic homeostasis, and disease progression [1,3,4]. Recent advances in high-throughput sequencing and metagenomics have enabled precise mapping of microbial composition and function [5], confirming that the GM not only participates in nutrient metabolism and pathogen defense [6,7] but also sustains intestinal barrier integrity by regulating epithelial renewal and differentiation [8,9]. Particularly noteworthy is the gut-brain axis, a bidirectional neuroendocrine-immune network that illustrates how microbial communities shape neurological conditions and systemic health outcomes through cross-organ communication [10]. These multilayered interactions demonstrate the GM’s regulatory role beyond the gut, whereas microbial metabolites such as short-chain fatty acids (SCFAs) further contribute to host adaptation by maintaining barrier function, modulating immune pathways, and influencing gene expression [1,3].

Emerging evidence indicates that microbial dysbiosis, characterized by reduced commensal abundance, proliferation of pathogenic taxa, and loss of ecological stability, exhibits significant associations with diverse public health challenges [1]. These encompass digestive disorders such as inflammatory bowel disease (IBD) [11-14] and functional bowel disorders [15,16]; systemic autoimmune conditions, including rheumatoid arthritis [17,18]; metabolic disturbances such as obesity [19] and glucose intolerance [20]; neurodegenerative pathologies such as Alzheimer disease [21] and Parkinson disease [22,23]; circadian rhythm disruption [24]; carcinogenic processes [25,26]; and neuropsychiatric manifestations, including mood disorders [10,27]. Mechanistic studies suggest that dysbiosis contributes to systemic pathology via endotoxemia, barrier dysfunction, and chronic inflammation. For instance, increased lipopolysaccharide exposure, impaired tight junctions, and nuclear factor kappa-light-chain-enhancer of activated B cells activation potentiate inflammation [28,29], whereas translocation of microbial products can induce apoptosis, oxidative stress, and systemic immune responses [30,31]. Recognition of microbial patterns by innate immune receptors further activates cytokine cascades, some of which cross the blood-brain barrier, potentially explaining microbe-driven neuropathology [10]. These mechanisms highlight the microbiome as both regulator and driver of systemic disease and support microbiota modulation as a promising preventive and therapeutic strategy [1,19,25].

Complementary and alternative therapies based on traditional Chinese medicine have increasingly been investigated for their capacity to reshape the GM [32,33]. Moxibustion, a thermal therapy using burning Artemisia argyi at acupoints along meridians, has shown potential to restore microbial balance and reduce inflammation. Compared with acupuncture, it requires minimal operator training, can be implemented by nurses at the bedside, and is well accepted by patients due to its noninvasive nature, making it cost-effective and feasible in resource-limited settings.

Preclinical and clinical evidence indicates that moxibustion can modulate microbial composition and diversity across multiple conditions. In a polycystic ovary syndrome rat model, moxibustion increased *Lactobacillus* abundance; reduced harmful taxa; and elevated SCFAs such as acetate, propionate, and butyrate, thereby improving the intestinal microenvironment [34]. In osteoarthritis models, treatment shifted microbiota profiles closer to those of healthy controls, significantly reducing *Ruminococcus* and *Proteobacteria* [35]. In ulcerative colitis, moxibustion alleviated dysbiosis and attenuated inflammation by downregulating NLRP3 inflammasome signaling [36,37]. Similarly, in colorectal cancer liver metastasis models, moxibustion reshaped disrupted microbiota and reduced metastatic progression [38]. Aging models further suggest that moxibustion enhances probiotic abundance and SCFA levels while suppressing proinflammatory mediators such as tumor necrosis factor (TNF) and interleukin-6 (IL-6) [39]. Collectively, these findings indicate that moxibustion may help restore microbial balance, improve barrier function, and reduce systemic inflammation.

Despite these promising observations, most reviews and meta-analyses have grouped acupuncture and moxibustion as a single intervention, limiting the ability to identify moxibustion’s unique contribution. A meta-analysis in 2024 [40] reported significant microbial shifts with combined “acupuncture-moxibustion,” including changes in *Firmicutes*, *Bacteroidetes*, and *Lactobacillus*, but did not distinguish each intervention’s distinct mechanisms. More recent studies have underscored that acupuncture and moxibustion differ substantially: acupuncture relies on mechanical needling and neural regulation, whereas moxibustion exerts effects capable of driving microbial remodeling via heat-dependent signaling pathways [41]. Importantly, acupuncture is frequently declined by patients due to needle-associated pain or anxiety and requires specialized medical training beyond the scope of nursing practice, whereas moxibustion has been formalized as a standardized traditional Chinese medicine nursing procedure [42]. Therefore, clarifying the exclusive effects of moxibustion on the GM is crucial for accurate mechanistic interpretation and for generating evidence-based recommendations in nursing and integrative medicine. This systematic review will synthesize clinical and preclinical evidence to evaluate moxibustion’s effects on microbial diversity, taxonomic composition, and functional metabolites and explore links between thermal dose parameters and microbiota-dependent outcomes.

By establishing standardized efficacy evaluation metrics and mechanistic frameworks, this study will advance the epistemological foundations of traditional medicine while generating tiered evidentiary support for policy implementation. The derived data will elucidate optimized protocols for microbiota-targeted therapies, facilitating their integration into multinational health care systems addressing microbiome-associated chronic diseases.

## Methods

### Study Registration

This protocol follows the PRISMA-P (Preferred Reporting Items for Systematic Review and Meta-Analysis Protocols) 2015 checklist [43]. Before commencement, the full methodological details were cataloged in the PROSPERO registry of planned systematic reviews (CRD42025639714).

### Ethical Considerations

The use of secondary data sources in this analytical study exempted it from institutional review board approval requirements. The finalized findings will be communicated to the scientific community through established academic channels, including journal manuscript submissions and international conference proceedings.

### Inclusion Criteria

#### Study Designs

This review will include clinical and preclinical studies assessing the effects of moxibustion on the GM. Observational studies, reviews, and case reports will be excluded. Only peer-reviewed articles will be considered, with a preference for studies demonstrating a low or moderate risk of bias to ensure the reliability and validity of the findings.

#### Study Types

Eligible studies must involve human participants or validated animal models evaluating GM outcomes, with a control group receiving no treatment, a placebo, or standard therapy.

In human studies, sham moxibustion may be used as a form of placebo control, defined as a procedure mimicking treatment without producing therapeutic effects (eg, nonacupoint application at ≥1.5 cm from recognized acupoints such as ST25, nonignited moxa sticks, or insulated devices). Standardized protocols are required, including consistent reporting of moxa type and stimulation duration, frequency, and distance. Eligible participants must have clinically confirmed GM-related disorders (eg, irritable bowel syndrome [IBS] via the Rome IV criteria; IBD through endoscopy; obesity via BMI≥30; or depression diagnosed via the *Diagnostic and Statistical Manual of Mental Disorders, Fifth Edition*, with Hamilton Depression Rating Scale scores of ≥17). Studies with patients with major comorbidities or recent antibiotic use will be excluded.

For animal studies, eligible experiments must use validated disease models (eg, dextran sulfate sodium [DSS]–induced colitis, high-fat diet–induced obesity, or chronic unpredictable mild stress–induced depression). Acceptable controls include no treatment, standard care, or sham procedures using nonignited moxa or equivalent thermal-neutral conditions under standardized dietary and housing conditions.

#### Interventions

This review will focus exclusively on moxibustion therapy, including direct, indirect, mild, or thunder-fire moxibustion, administered either as a stand-alone intervention or in combination with adjunctive treatments. Where combination therapies are applied (eg, moxibustion plus mesalamine for IBD), the same adjunctive regimen must be provided to both the intervention and control groups. To ensure comparability, details such as drug type, dosage, treatment duration, and adherence should be reported; studies lacking such information will be flagged and considered in sensitivity analyses. Studies involving probiotics or unmatched cointerventions will be excluded.

### Exclusion Criteria

Studies will be excluded if they meet any of the following criteria:

1. Nonvalidated in vitro or ex vivo studies: in vitro (eg, cell culture) or ex vivo experiments lacking in vivo validation (animal models or human trials) to confirm GM outcomes
2. Nonprimary research: reviews, meta-analyses, case reports, conference abstracts, editorials, or expert opinions (due to inability to extract original data)
3. Noninterventional designs: case studies, surveys, or purely computational (in silico) analyses without experimental validation
4. Irrelevant outcomes: studies lacking quantitative GM metrics (eg, 16S ribosomal RNA [rRNA] sequencing, metagenomic analysis, α or β diversity, or taxa abundance changes)
5. Data deficiencies: duplicate publications (the most comprehensive dataset will be retained; preprints will be excluded if peer-reviewed versions exist) or insufficient data for meta-analysis (eg, missing mean or SD, sample size, or statistical comparability between groups)

### Primary Outcome

The primary outcomes will be changes in GM diversity (Shannon index and Simpson index) and relative abundance of major bacterial phyla (*Firmicutes* and *Bacteroidetes*) and beneficial genera (*Lactobacillus* and *Bifidobacterium*).

### Secondary Outcomes

The secondary outcomes will be levels of SCFAs, inflammatory markers (eg, IL-6 and TNF), and clinical symptom improvement scores.

### Search Strategy

A comprehensive literature search will be conducted across the PubMed, Web of Science, Cochrane Library, China National Knowledge Infrastructure, Wanfang, and VIP databases from inception to December 31, 2024. The search will use a combination of MeSH (Medical Subject Heading) terms and free-text keywords pertinent to moxibustion and the GM, including terms such as “moxibustion,” “moxa therapy,” “gut microbiota,” and “intestinal flora.” The strategy will follow PRISMA-S (PRISMA literature search extension) guidelines. In addition, the reference lists of relevant studies and systematic reviews will be manually screened to identify any further eligible studies. No language restrictions will be applied during the initial search. Studies published in Chinese will be included if they meet the eligibility criteria, with independent translation and data extraction performed by 2 bilingual reviewers. Discrepancies will be resolved through consensus or adjudication by a third reviewer. Non–English-language studies will be screened at the title or abstract level and, if potentially eligible, translated using professional services or validated translation software to ensure accurate data extraction. A detailed description of the search strategy is provided in Multimedia Appendix 1.

### Study Selection

A blinded evaluation system will be implemented where 2 researchers will independently analyze bibliographic records through the NoteExpress digital workflow management software. Initial screening of metadata will progress to full-text appraisal, with interrater inconsistencies addressed through consensus-building dialogues or adjudication. The study selection process will be visually mapped using established PRISMA diagrammatic conventions.

### Data Extraction

Two independent reviewers will extract data using a standardized, pilot-tested form (Multimedia Appendix 2). For human studies, details will include participant demographics (age and gender), diagnostic criteria (eg, Rome IV for IBS), and clinical settings; for animal studies, species and strain, disease induction protocols (eg, 5% DSS for colitis), and environmental controls (diet and housing) will be recorded. Moxibustion parameters (type [direct, indirect, or mild], acupoints using World Health Organization nomenclature [eg, ST25 or “Tianshu”], and session duration and frequency) and microbiome sequencing details (platform: Illumina NovaSeq; 16S rRNA region: V3-V4; bioinformatics pipeline: QIIME 2 with SILVA version 138) will be systematically extracted. To minimize heterogeneity, sequencing parameters (platform, primer region, reference database, and pipeline version) will be recorded in detail, and subgroup analyses will be conducted based on these factors. GM outcomes include α diversity indexes (Shannon, Simpson, and Chao1) and β diversity metrics (Bray-Curtis permutational multivariate ANOVA) alongside taxonomic abundances (phylum and genus level). Where raw or normalized abundance data are reported, potential batch effects will be noted; if feasible, batch correction strategies (eg, ComBat and remove unwanted variation) will be documented during extraction. Missing data will be retrieved via WebPlotDigitizer (version 4.6) or author correspondence (2 email attempts, 14-day interval). Multiple treatment arms will be analyzed separately. Data will be standardized (relative abundance conversion and log transformation) and archived in formats compliant with the findability, accessibility, interoperability, and reusability principles (CSV and Microsoft Excel) on Figshare (Digital Science). Interreviewer consistency will be validated using the Cohen κ (threshold of ≥0.8), with discrepancies resolved by a third reviewer.

### Assessment of Risk of Bias

The risk of bias will be evaluated using a modified version of the Systematic Review Center for Laboratory Animal Experimentation risk-of-bias tool [44] and the Collaborative Approach to Meta-Analysis and Review of Animal Data from Experimental Studies checklist [40,45]. The assessment will cover the following domains: (1) adequacy of random sequence generation, (2) baseline comparability between groups or adjustment for confounding variables, (3) allocation concealment, (4) random housing of animals during the experiment (for animal studies only), (5) blinding of caregivers and researchers, (6) random selection of participants for outcome assessment, (7) blinding of outcome assessors, (8) handling of incomplete outcome data, (9) selective outcome reporting, and (10) presence of other potential sources of bias. The same checklist will be applied for human studies except for domain 4, which is not applicable for these studies.

Each item will be rated as having a low, high, or unclear risk of bias. Although complete blinding of moxibustion interventions is challenging, outcome assessment blinding will still be evaluated. Any disagreements in bias assessment will be resolved through discussion or consultation with a third reviewer.

### Data Synthesis

Meta-analyses will be conducted when ≥5 studies report comparable GM outcomes (eg, α diversity and Firmicutes-to-Bacteroidetes ratio) to ensure statistical power following PRISMA-P (PRISMA-Protocols) guidelines. For continuous variables such as Shannon or Simpson indexes and *Firmicutes/Bacteroidetes* ratios, values will be pooled using the ratio of means with 95% CIs, calculated by dividing the treatment group mean by the control group mean [40,46]. To address skewed distributions common in microbiome data (eg, relative abundance), arcsine square root transformation will stabilize the variance by reducing the disproportionate influence of extreme proportions, whereas log transformation will normalize metrics such as SCFA concentrations to approximate Gaussian distribution and enable valid parametric comparisons. Zero-inflated distributions of low-abundance taxa will be addressed by applying centered log ratio transformation, aggregating rare taxa to higher taxonomic levels (family and phylum), and excluding features present in <10% of the samples in sensitivity analyses. A random-effects model (DerSimonian-Laird estimator) will prioritize heterogeneity adjustments arising from variations in study designs (eg, randomized controlled trials vs animal models), sequencing platforms (eg, Illumina vs Ion Torrent), primer regions (eg, V3-V4 vs V4-V5), bioinformatics pipelines (eg, QIIME 2 vs Mothur), and intervention protocols (eg, direct vs indirect moxibustion).

Heterogeneity will be quantified using the Cochran *Q* test (α=.10) and the *I*^2^ statistic (*I*^2^≥75% indicating substantial heterogeneity). For secondary outcomes (SCFAs and inflammatory markers), standardized mean differences will harmonize heterogeneous units (eg, mmol/L vs μg/g). Both clinical and preclinical studies will be included in the primary meta-analysis to maximize statistical power and identify overall patterns. To further address heterogeneity, subgroup analyses will be conducted by study type (clinical vs animal), disease category (IBD, IBS, and obesity), model type (eg, DSS-induced colitis vs chronic unpredictable mild stress–induced depression), and sequencing platform. In addition, protocol-related variables will be explored: subgroup analyses will be conducted by moxibustion type (direct, indirect, mild, and thunder-fire), treatment duration (≤4 weeks vs >4 weeks), and session frequency (≤3 vs >3 sessions per week). Where substantial heterogeneity persists, narrative synthesis will complement quantitative findings. Sensitivity analyses will exclude studies with high risk of bias or nonstandardized methodologies (eg, quantitative polymerase chain reaction–only data or incomplete sequencing metadata). Batch effect correction will be attempted in sensitivity analyses when raw abundance data are available. All analyses will be conducted in RevMan (version 5.4; The Cochrane Collaboration) and validated using R (*metafor* package; R Foundation for Statistical Computing), with scripts and datasets archived on Figshare to ensure reproducibility. *P*<.05 will be considered statistically significant for the overall effect.

### Publication Bias and Evidence Quality

If ≥10 studies are included, contour-enhanced funnel plots and the Egger test (*P*<.05) [47] will assess publication bias. The Grading of Recommendations Assessment, Development, and Evaluation system [48] will evaluate evidence quality, downgrading for risk of bias, inconsistency (*I*^2^>50%), indirectness (eg, animal-to-human extrapolation), and imprecision (wide CIs).

## Results

**Figure 1.**
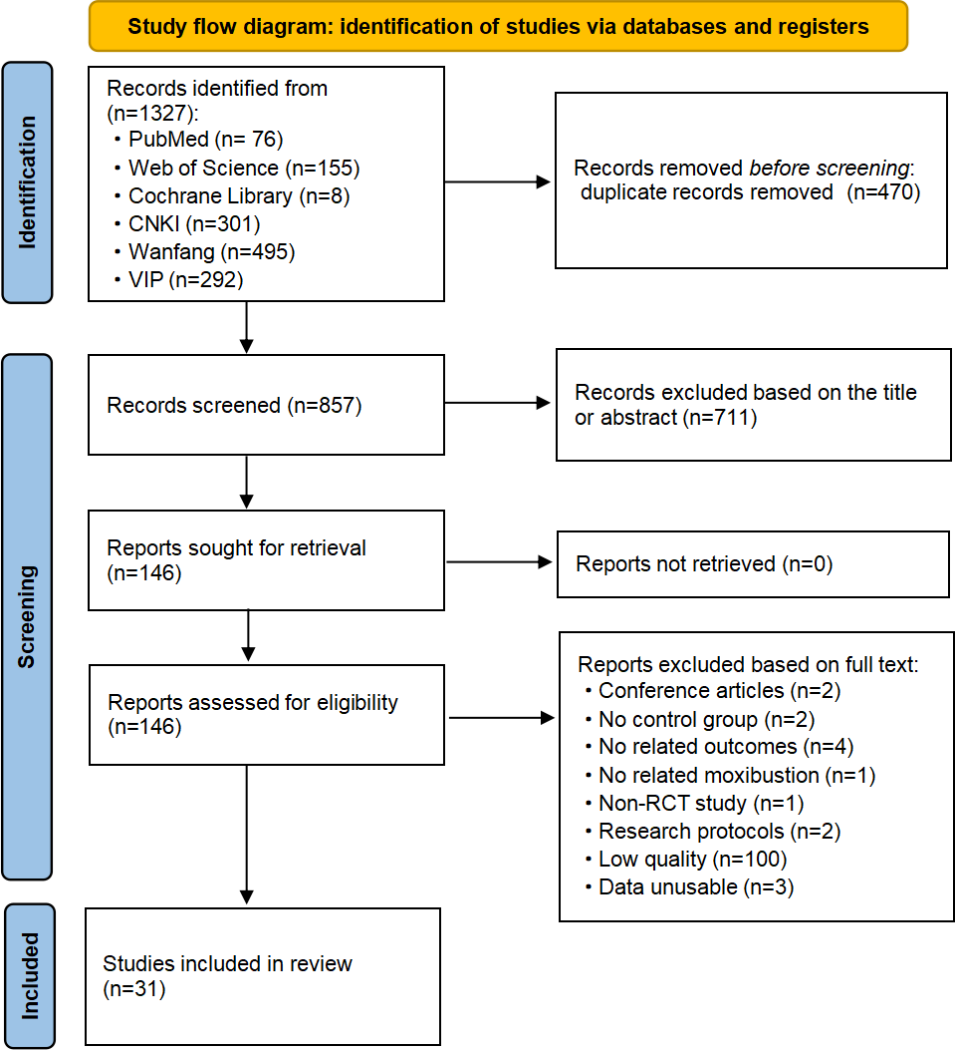
Study flow diagram. CNKI: China National Knowledge Infrastructure; RCT: randomized controlled trial.

The protocol was funded by the 2024 Nanjing University of Chinese Medicine Natural Science Foundation (grant XZR2024009) in March 2024. We identified 1327 records across 6 databases, of which 470 duplicates were removed (470/1327, 35.4%). After screening 857 titles and abstracts, 146 full texts were reviewed (146/857, 17.0%), and 31 studies met the eligibility criteria (31/857, 3.6%) for inclusion (Figure 1). The review is progressing through quality assessment and data synthesis, with final analyses expected by October 2025. Final results are anticipated by late 2025.

## Discussion

The GM constitutes an indispensable microbial community within the human physiological system. Research on the gut-brain axis confirms that the GM mediates bidirectional interactions among neural, metabolic, and immune systems, extending its influence beyond gastrointestinal functions [49]. Studies have demonstrated that microbial community stability exhibits sensitivity to host-intrinsic variables (genetic predisposition, gestational changes, and aging) and environmental modulators (dietary patterns, antibiotic exposure, and behavioral factors) [50]. Pathological alterations in microbiota composition (dysbiosis) show close associations with aberrant adaptive immune activation, sustaining chronic inflammatory states that may constitute the pathological basis for various diseases, including degenerative joint cartilage lesions [51] and IBD [52]. Current therapeutic paradigms increasingly recognize microbial ecosystem engineering as a viable intervention strategy, with microbiota-targeted therapies demonstrating clinical translational potential. The mechanistic foundation of such interventions lies in restoring microbial diversity and functional redundancy, thereby re-establishing host-microbe symbiosis that is critical for physiological equilibrium.

Moxibustion, a therapeutic modality rooted in traditional medicine, regulates homeostatic processes through thermal stimulation generated by burning *Artemisia argyi* at specific acupoints or meridian pathways. This intervention has gained recognition for its immunomodulatory properties and capacity to regulate inflammatory pathways, with emerging evidence suggesting its potential to exert broader systemic effects via gut microbiome modulation. Contemporary research demonstrates that moxibustion’s therapeutic spectrum extends beyond analgesia and immune system optimization to encompass gut microbial community restructuring. Notably, experimental models reveal its ability to enhance commensal microbiota while suppressing pathogenic genera, thereby promoting intestinal ecological equilibrium and mitigating inflammatory cascades [34]. This multimodal therapeutic profile positions moxibustion as a viable treatment option for patients with pharmacotherapy contraindications or preferences for noninvasive interventions. While preliminary data suggest that microbiota-mediated mechanisms may contribute to moxibustion’s clinical effects, its precise biomolecular pathways and long-term therapeutic outcomes require systematic exploration. Current knowledge gaps underscore the necessity for rigorously designed clinical trials coupled with mechanistic studies integrating multiomics analyses. Such investigations could elucidate the intervention’s pleiotropic actions while establishing evidence-based protocols to facilitate its integration into modern therapeutic paradigms.

This systematic review protocol provides a framework to evaluate the exclusive effects of moxibustion on GM modulation. By focusing on microbial diversity, taxonomic shifts, and metabolites (eg, SCFAs), this study bridges traditional medicine with modern microbiome science. The protocol’s emphasis on sham-controlled trials, standardized moxibustion parameters (eg, acupoint localization and session duration), and consistent handling of combination therapies enhances methodological rigor and comparability. Preclinical studies [35] provide preliminary evidence that moxibustion may reduce pathogenic taxa (*Ruminococcus* and *Proteobacteria*) and increase beneficial microbes (*Lactobacillus* and *Bifidobacterium*), correlating with improved barrier integrity and reduced inflammatory markers (TNF and IL-6). These findings align with data linking GM regulation to outcomes in osteoarthritis and IBD, where cyclic adenosine monophosphate signaling pathways may mediate anti-inflammatory effects.

Nevertheless, the causal direction of these associations remains uncertain. Moxibustion may act directly on host physiology—through immune regulation, neuroendocrine signaling, or circulatory changes—with secondary effects on the GM, or primary microbiota shifts may instead drive systemic improvements. A bidirectional relationship is also plausible, consistent with the gut-brain and gut-immune axes. These possibilities will be examined in this review. Although causality cannot be established, this review will identify consistent patterns and propose mechanistic hypotheses to guide future longitudinal and interventional microbiome studies. By integrating clinical and preclinical models, this review will clarify whether moxibustion-induced GM changes are consistent across species and pathologies, supporting its potential as a scalable, nonpharmacological intervention for microbiome-associated disorders.

Despite its strengths, several limitations warrant consideration. First, variability in moxibustion protocols (eg, direct vs indirect methods, acupoint selection, and treatment duration) and microbiome profiling techniques (eg, 16S rRNA variable regions, sequencing platforms, and bioinformatics pipelines) may complicate cross-study comparisons. Although random-effects models and subgroup analyses will be applied, residual confounding from unmeasured factors such as host genetics or dietary habits may persist. Second, publication bias remains possible as negative findings in traditional medicine research are often underreported. Funnel plots and Egger tests will be used to evaluate this risk. Third, achieving complete blinding is inherently difficult for moxibustion in clinical settings. Even with sham devices, participants may perceive differences in heat intensity or treatment location, introducing expectation bias. Although this will be addressed by assessing blinding quality and conducting sensitivity analyses, residual performance bias cannot be entirely ruled out. Finally, this review made the decision to pool both clinical and preclinical studies in the primary analysis. While this approach increases statistical power and enables broader pattern recognition, it introduces potential cross-species heterogeneity because animal models may not fully replicate human physiology or microbiota composition. Although subgroup analyses by study type and disease or model category will be conducted to explore sources of heterogeneity, residual differences between clinical and preclinical evidence may limit the direct generalizability of pooled results to patient populations. These factors will be explicitly acknowledged when interpreting the findings.

Thus, future studies should prioritize dose-response optimization to define thermal parameters (intensity, duration, and frequency) that maximize GM modulation while minimizing adverse effects. Furthermore, exploring synergistic therapies combining moxibustion with probiotics or dietary interventions could amplify SCFA-driven barrier restoration.

## Supplementary material

10.2196/73317 Multimedia Appendix 1 Search strategy for the systematic review of the exclusive effects of moxibustion on gut microbiota.

10.2196/73317 Multimedia Appendix 2 Data extraction framework for studies on the exclusive effects of moxibustion on gut microbiota.

10.2196/73317 Checklist 1 PRISMA-P checklist.

## Abbreviations

DSS: dextran sulfate sodium
GM: gut microbiota
IBD: inflammatory bowel disease
IBS: irritable bowel syndrome
IL-6: interleukin-6
MeSH: Medical Subject Heading
PRISMA: Preferred Reporting Items for Systematic Reviews and Meta-Analyses
PRISMA-P: Preferred Reporting Items for Systematic Reviews and Meta-Analyses–Protocols
PRISMA-S: Preferred Reporting Items for Systematic Reviews and Meta-Analyses literature search extension
rRNA: ribosomal ribonucleic acid
SCFA: short-chain fatty acid
TNF: tumor necrosis factor
