# Supplementary material for: Exclusive Effects of Moxibustion on Gut Microbiota: Protocol for a Focused Systematic Review and Meta-Analysis
Source: JMIR Res Protoc. 2025 Oct 24;14:e73317. doi: 10.2196/73317 (PMC12551972; doi:10.2196/73317)
Supplement: Multimedia Appendix 1 [file resprot-v14-e73317-s001.docx]

| **Database** | **Search**  **number** | **Query** | **Results** |
| --- | --- | --- | --- |
| PubMed | #1 | "Moxibustion"[MeSH Terms] | 3011 |
|  | #2 | "Moxabustion"[Title/Abstract] OR "Moxa"[Title/Abstract] OR "Mugwort"[Title/Abstract] OR "Wormwood"[Title/Abstract] OR "Artemisia"[Title/Abstract] | 6967 |
|  | #3 | #1 OR #2 | 9652 |
|  | #4 | "Gastrointestinal Microbiome"[Mesh] | 51223 |
|  | #5 | (((((((((((((((((((((((((((((((((((((Gastrointestinal Microbiome[Title/Abstract]) OR (Gastrointestinal Microbiomes[Title/Abstract])) OR (Microbiome, Gastrointestinal[Title/Abstract])) OR (Gut Microbiome[Title/Abstract])) OR (Gut  Microbiomes[Title/Abstract])) OR (Microbiome, Gut[Title/Abstract])) OR (Gut Microflora[Title/Abstract])) OR (Microflora, Gut[Title/Abstract])) OR (Gut Microbiota[Title/Abstract])) OR (Gut Microbiotas[Title/Abstract])) OR (Microbiota, Gut[Title/Abstract])) OR (Gastrointestinal Flora[Title/Abstract])) OR (Flora, Gastrointestinal[Title/Abstract])) OR (Gut Flora[Title/Abstract])) OR (Flora, Gut[Title/Abstract])) OR (Gastrointestinal Microbiota[Title/Abstract])) OR (Gastrointestinal Microbiotas[Title/Abstract])) OR (Microbiota, Gastrointestinal[Title/Abstract])) OR (Gastrointestinal Microbial Community[Title/Abstract])) OR (Gastrointestinal Microbial Communities[Title/Abstract])) OR (Microbial Community, Gastrointestinal[Title/Abstract])) OR (Gastrointestinal Microflora[Title/Abstract])) OR (Microflora, Gastrointestinal[Title/Abstract])) OR (Gastric Microbiome[Title/Abstract])) OR (Gastric Microbiomes[Title/Abstract])) OR (Microbiome, Gastric[Title/Abstract])) OR (Intestinal Microbiome[Title/Abstract])) OR (Intestinal Microbiomes[Title/Abstract])) OR (Microbiome, Intestinal[Title/Abstract])) OR (Intestinal Microbiota[Title/Abstract])) OR (Intestinal Microbiotas[Title/Abstract])) OR (Microbiota, Intestinal[Title/Abstract])) OR (Intestinal Microflora[Title/Abstract])) OR (Microflora, Intestinal[Title/Abstract])) OR (Intestinal Flora[Title/Abstract])) OR (Flora, Intestinal[Title/Abstract])) OR (Enteric Bacteria[Title/Abstract])) OR (Bacteria, Enteric[Title/Abstract]) | 102080 |
|  | #6 | #4 OR #5 | 110029 |
|  | #7 | #3 AND #6 | 76 |
|  | #8 | #7 AND (1000/1/1:2024/12/31[pdat]) | 76 |
| Web of science | #1 | TS=(Moxabustion OR Moxa OR Mugwort OR Wormwood OR Artemisia) | 25095 |
|  | #2 | TS=(Gastrointestinal Microbiome OR Gastrointestinal Microbiomes OR Microbiome, Gastrointestinal OR Gut Microbiome OR Gut Microbiomes OR Microbiome, Gut OR Gut Microflora OR Microflora, Gut OR Gut Microbiota OR Gut Microbiotas OR Microbiota, Gut OR Gastrointestinal Flora OR Flora, Gastrointestinal OR Gut Flora OR Flora, Gut OR Gastrointestinal Microbiota OR Gastrointestinal Microbiotas OR Microbiota, Gastrointestinal OR Gastrointestinal Microbial Community OR Gastrointestinal Microbial Communities OR Microbial Community, Gastrointestinal OR Gastrointestinal Microflora OR Microflora, Gastrointestinal OR Gastric Microbiome OR Gastric Microbiomes OR Microbiome, Gastric OR Intestinal Microbiome OR Intestinal Microbiomes OR Microbiome, Intestinal OR Intestinal Microbiota OR Intestinal Microbiotas OR Microbiota, Intestinal OR Intestinal Microflora OR Microflora, Intestinal OR Intestinal Flora OR Flora, Intestinal OR Enteric Bacteria OR Bacteria, Enteric) | 220687 |
|  | #3 | #1 AND #2 | 155 |
|  |  | Limiters applied:   1. Publication Date: 2000-01-01 to 2024-12-31   https://webofscience.clarivate.cn/wos/alldb/summary/7052a8bc-8abf-4cb5-ae48-5bd2c1abed07-0144f33660/relevance/1 | 155 |
| Cochrane Library | #1 | MeSH descriptor: [Moxibustion] explode all trees | 697 |
|  | #2 | (Moxabustion):ti,ab,kw OR (Moxa):ti,ab,kw OR (Mugwort):ti,ab,kw OR (Wormwood):ti,ab,kw OR (Artemisia):ti,ab,kw | 511 |
|  | #3 | #1 OR #2 | 1118 |
|  | #4 | MeSH descriptor: [Gastrointestinal Microbiome] explode all trees | 1898 |
|  | #5 | (Gastrointestinal Microbiome):ti,ab,kw OR (Gastrointestinal Microbiomes):ti,ab,kw OR (Microbiome, Gastrointestinal):ti,ab,kw OR (Gut Microbiome):ti,ab,kw OR (Gut Microbiomes):ti,ab,kw | 4582 |
|  | #6 | (Microbiome, Gut):ti,ab,kw OR (Gut Microflora):ti,ab,kw OR (Microflora, Gut):ti,ab,kw OR (Gut Microbiota):ti,ab,kw OR (Gut Microbiotas):ti,ab,kw | 7439 |
|  | #7 | (Microbiota, Gut):ti,ab,kw OR (Gastrointestinal Flora):ti,ab,kw OR (Flora, Gastrointestinal):ti,ab,kw OR (Gut Flora):ti,ab,kw OR (Flora, Gut):ti,ab,kw | 6777 |
|  | #8 | (Gastrointestinal Microbiota):ti,ab,kw OR (Gastrointestinal Microbiotas):ti,ab,kw OR (Microbiota, Gastrointestinal):ti,ab,kw OR (Gastrointestinal Microbial  Community):ti,ab,kw OR (Gastrointestinal Microbial Communities):ti,ab,kw | 3101 |
|  | #9 | (Microbial Community, Gastrointestinal):ti,ab,kw OR (Gastrointestinal Microflora):ti,ab,kw OR (Microflora, Gastrointestinal):ti,ab,kw OR (Gastric Microbiome):ti,ab,kw OR (Gastric Microbiomes):ti,ab,kw | 1084 |
|  | #10 | (Microbiome, Gastric):ti,ab,kw OR (Intestinal Microbiome):ti,ab,kw OR (Intestinal Microbiomes):ti,ab,kw OR (Microbiome, Intestinal):ti,ab,kw OR (Intestinal Microbiota):ti,ab,kw | 3498 |
|  | #11 | (Intestinal Microbiotas):ti,ab,kw OR (Microbiota, Intestinal):ti,ab,kw OR (Intestinal Microflora):ti,ab,kw OR (Microflora, Intestinal):ti,ab,kw OR (Intestinal Flora):ti,ab,kw | 4623 |
|  | #12 | (Flora, Intestinal):ti,ab,kw OR (Enteric Bacteria):ti,ab,kw OR (Bacteria, Enteric):ti,ab,kw | 2832 |
|  | #13 | #4 OR #5 OR #6 OR #7 OR #8 OR #9 OR #10 OR #11 OR #12 | 11726 |
|  | #14 | #3 AND #13 | 8 |
| CNKI |  | (SU%'艾灸' + '灸法' + '灸') and (SU%'肠道微生物' + '肠道菌群' + '肠道微生态') | 303 |
|  |  | 时间范围：至2024-12-31 | 301 |
| Wanfang Data |  | 主题= (艾灸 OR 灸法 OR 灸) AND 主题= (肠道微生物 OR 肠道菌群 OR 肠道微生态) | 495 |
|  |  | 发表时间：不限-2024年 | 495 |
| VIP |  | U=(艾灸 OR 灸法 OR 灸) AND U=(肠道微生物 OR 肠道菌群 OR 肠道微生态) | 292 |
|  |  | 时间范围：2000-01至2024-12 | 292 |
