# Supplementary material for: Exclusive Effects of Moxibustion on Gut Microbiota: Protocol for a Focused Systematic Review and Meta-Analysis
Source: JMIR Res Protoc. 2025 Oct 24;14:e73317. doi: 10.2196/73317 (PMC12551972; doi:10.2196/73317)
Supplement: Multimedia Appendix 2 [file resprot-v14-e73317-s002.docx]

**Table 2:** Data extraction framework for studies on the exclusive effects of moxibustion on gut microbiota.

| Author, year (country) | Disease/Model | Vendor/Hospital | Sample size (T/C) | Interventionprotocols (T/C) | Type of MOX | | | | Microbiome Sequencing | | | | Gut microbiota relative outcome | | | SCFAs | Inflamm-atory markers | Clinical symptom improve-ment scores |
| --- | --- | --- | --- | --- | --- | --- | --- | --- | --- | --- | --- | --- | --- | --- | --- | --- | --- | --- |
|  |  |  |  |  | Acupoint(s) | Parameter(s)  (Depth/Distance) | Time | Number/Duration of Treatment | Platform | Strategy | Variable Region | Extraction Method | α- diversity | β-diversity | The relative abundances on phylum and genus levels |  |  |  |
|  |  |  |  |  |  |  |  |  |  |  |  |  |  |  |  |  |  |  |
|  |  |  |  |  |  |  |  |  |  |  |  |  |  |  |  |  |  |  |
|  |  |  |  |  |  |  |  |  |  |  |  |  |  |  |  |  |  |  |
|  |  |  |  |  |  |  |  |  |  |  |  |  |  |  |  |  |  |  |

Notes: T: Treatment groups; C: Control groups; MOX: Moxibustion
